# Comparative Transcriptomics of Strawberries (*Fragaria* spp.) Provides Insights into Evolutionary Patterns

**DOI:** 10.3389/fpls.2016.01839

**Published:** 2016-12-15

**Authors:** Qin Qiao, Li Xue, Qia Wang, Hang Sun, Yang Zhong, Jinling Huang, Jiajun Lei, Ticao Zhang

**Affiliations:** ^1^School of Agriculture, Yunnan UniversityKunming, China; ^2^College of Horticulture, Shenyang Agricultural UniversityShenyang, China; ^3^Key Laboratory for Plant Diversity and Biogeography of East Asia, Kunming Institute of Botany, Chinese Academy of SciencesKunming, China; ^4^Key Laboratory for Biodiversity Science and Ecological Engineering, School of Life Sciences, Fudan UniversityShanghai, China; ^5^Department of Biology, East Carolina University, GreenvilleNC, USA

**Keywords:** *Fragaria*, transcriptome, evolutionary pattern, comparative genomics, positive selection

## Abstract

Multiple closely related species with genomic sequences provide an ideal system for studies on comparative and evolutionary genomics, as well as the mechanism of speciation. The whole genome sequences of six strawberry species (*Fragaria* spp.) have been released, which provide one of the richest genomic resources of any plant genus. In this study, we first generated seven transcriptome sequences of *Fragaria* species *de novo*, with a total of 48,557–82,537 unigenes per species. Combined with 13 other species genomes in Rosales, we reconstructed a phylogenetic tree at the genomic level. The phylogenic tree shows that *Fragaria* closed grouped with *Rubus* and the *Fragaria* clade is divided into three subclades. East Asian species appeared in every subclade, suggesting that the genus originated in this area at ∼7.99 Mya. Four species found in mountains of Southwest China originated at ∼3.98 Mya, suggesting that rapid speciation occurred to adapt to changing environments following the uplift of the Qinghai–Tibet Plateau. Moreover, we identified 510 very significantly positively selected genes in the cultivated species *F*. × *ananassa* genome. This set of genes was enriched in functions related to specific agronomic traits, such as carbon metabolism and plant hormone signal transduction processes, which are directly related to fruit quality and flavor. These findings illustrate comprehensive evolutionary patterns in *Fragaria* and the genetic basis of fruit domestication of cultivated strawberry at the genomic/transcriptomic level.

## Introduction

Multiple closely related species with genomic sequences provide an ideal system for studies on comparative and evolutionary genomics, as well as the mechanism of speciation. However, only a few genera in eukaryotes, e.g., *Drosophila* (Clark et al., 2007) and *Oryza* (Zhang et al., 2014), contain multiple genome sequenced relatives because it is expensive to sequence whole genomes at present. Fortunately, transcriptome sequencing (using RNA-seq technology) provides a rapid and effective approach to obtain large numbers of protein-coding genes, which can be used for understanding evolutionary questions in non-model organisms (Ward et al., 2012; Haas et al., 2013). Recently, comparative transcriptome studies between multiple close relatives have been used to reveal evolutionary patterns in domesticated and wild tomato (Koenig et al., 2013), and for phylogenomic analysis in Caryophyllales (Yang et al., 2015).

The genus *Fragaria* L. (Rosaceae) contains about 25 species, which are most spring-blooming, insect-pollinated herbaceous perennials capable of clonal growth, and have animal-dispersed fleshy fruits (Johnson et al., 2014; Liston et al., 2014). This genus has diversified mating systems and ploidy (2×, 4×, 6×, 8×, and 10×; Liston et al., 2014). Cultivated strawberry, *F*. × *ananassa* (2n = 8× = 56), is an important fruit crop, which originated via octoploid hybridization between *F. virginiana* and *F. chiloensis* in 18th century in Europe. Like natural selection, domestication has influenced the evolutionary pressures on the cultivated strawberry. The geographic distribution of *Fragaria* spans a wide range of Eurasia, North and South America and East Asia (Johnson et al., 2014; Liston et al., 2014). Of these, East Asia is the center of genetic diversity and the most likely original range of *Fragaria* (Njuguna et al., 2013). In addition, dated molecular phylogenies based on whole chloroplast genomes indicated that the genus is quite young (1.0–4.1 Mya; Njuguna et al., 2013). Obviously, this younger genus must have undergone rapid spread and species differentiation to adapt to different environments.

Previous studies have obtained the whole genome sequences of one cultivated (*F*. × *ananassa*) and five wild strawberries (*F. vesca*, *F. iinumae*, *F. nipponica*, *F. nubicola*, and *F. orientalis*; Shulaev et al., 2011; Hirakawa et al., 2014), which provide one of the richest genomic resources of any plant genus. Most diploid species in *Fragaria* have very small genomes (∼200 Mb, **Table 1**) compared to other higher plants. Recently evidence from linkage maps method indicated that four subgenomes of the allo-octoploid cultivated strawberry *F*. × *ananassa* were originated with diploid donors of *F. vesca*, *F.iinumae* and two *F. iinumae*-like ancestors (Tennessen et al., 2014; Sargent et al., 2016). A virtual diploid reference genome (173.2 Mb, contains 45,377 genes) of the cultivated strawberry was constructed by integrating the sequences of the four homoeologous subgenomes of *F*. × *ananassa* (in total 697.8 Mb, contains 230,838 genes), which eliminated both heterozygous regions as well as homeologous differences (i.e., differences between subgenomes) using overlap graph method, Hirakawa et al., 2014). Except for *Fragaria*, the genome sequences of other important Rosaceae fruit crops are also currently available, e.g., apple (*Malus domestica*), pear (*Pyrus communis*), peach (*Prunus persica*), Chinese plum (*P. mume*) and black raspberry (*Rubus occidentalis*). These abundant genome resources make it possible to improve our understanding of the genetics of horticultural traits by comparative genomics, including fruit quality, timing of flowering, and pest and disease resistance. In short, *Fragaria* is a very attractive model genus for the study of recent speciation, molecular ecology, evolutionary genomics and fruit development (Liston et al., 2014).

**Table 1:** Statistics of study materials and gene numbers in *Fragaria*.

Species	Abbreviation	Ploidy	Mating system	Genome size (Mb)	Number of genes	Reference
*F. vesca*	FVE	2*x*	Hermaphrodite, SC	220.2	34809	Shulaev et al., 2011
*F. nipponica*	FNI	2*x*	Hermaphrodite, SI	206.4	87803	Hirakawa et al., 2014
*F. iinumae*	FII	2*x*	Hermaphrodite, SC	199.6	76760	Hirakawa et al., 2014
*F. nubicola*	FNU	2*x*	Hermaphrodite, SI	203.7	85062	Hirakawa et al., 2014
*F. nilgerrensis*	FNG	2*x*	Hermaphrodite, SC	274 ± 3.8^∗^	82537	This study
*F. pentaphylla*	FPE	2*x*	Hermaphrodite, SI	268 ± 3.7^∗^	53748	This study
*F. mandshurica*	FMA	2*x*	Hermaphrodite, SI	241 ± 4.3^∗^	55911	This study
*F. viridis*	FVI	2*x*	Hermaphrodite, SI	230 ± 2.8^∗^	48557	This study
*F. orientalis*	FOR	4*x*	Dioecious	214.2	99674	Hirakawa et al., 2014
*F. corymbosa*	FCO	4*x*	Dioecious	NA	62587	This study
*F. moupinensis*	FMO	4*x*	Dioecious	245 ± 3.3^∗^	52413	This study
*F. tibetica*	FTI	4*x*	Dioecious	NA	54022	This study
*F.* × *ananassa*	–	8*x*	Subdioecious	697.8	230838	Hirakawa et al., 2014
FANhybrid	FAN	2*x* (virtual diploid genome)		173.2	45377	Hirakawa et al., 2014


*SC, self-compatible; SI, self-incompatible. ^∗^referred from Chen et al. (2015).*

In this study, we performed RNA-seq technology to obtain most transcript sequences of seven diploid and tetraploid *Fragaria* species (**Tables 1** and **2**). Combining these with six *Fragaria* species genomes (including the virtual diploid genome of *F*. × *ananassa*) and seven other species which also have sequenced genomes in Rosales, we first reconstructed a phylogenetic tree based on single copy genes at the genomic level and estimated the divergence time of each node. Subsequently, the substitution rates were estimated in eight diploid strawberries to identify the rate of evolution in each species. Finally, positively selected genes (PSGs) related to agronomic characters in the cultivated strawberry were identified using evolutionary genomics. We aim to gain a comprehensive understanding of the evolutionary patterns in *Fragaria* at the genomic/transcriptomic level.

## Materials and Methods

### Sample Collection and Transcriptome Sequencing

Seven wild strawberries, i.e., *F. nilgerrensis* (FNG), *F. pentaphylla* (FPE), *F. mandshurica* (FMA), *F. viridis* (FVI), *F. corymbosa* (FCO), *F. moupinensis* (FMO), and *F. tibetica* (FTI), which collected from East Asia were cultivated in cultivation base of Shenyang Agricultural University. In order to obtain more expression transcripts, different organs (leaves, stems, flowers, and fruits) of each species were sampled and stored at -80°C. Among these species, except the FNG were sequenced using two individuals separately, other six species were sequenced using one individual with pooled different tissues.

High quality total RNA was extracted using the TRIZOL reagent (Sigma-Aldrich) following the manufacturer’s instructions. A total amount of 3 μg RNA per species was used as input material for the RNA sample preparations. Sequencing libraries were generated using NEBNext Ultra^TM^ RNA Library Prep Kit for Illumina Inc. (NEB, USA) following manufacturer’s recommendations. The clustering of the index-coded samples was performed on a cBot Cluster Generation System using TruSeq PE Cluster Kit v3-cBot-HS (Illumina Inc.). After cluster generation, the library preparations were sequenced on an Illumina Hiseq 2500 platform and paired-end reads were generated. The whole step of library construction and Illumina sequencing was performed at Novogene Bioinformatics Technology Co., Ltd (Beijing, China).

### *De novo* Assembly and Functional Annotation

Clean data were obtained by removing reads containing adapter, reads containing ploy-N and low quality reads from raw data. At the same time, Q20, Q30, GC-content and sequence duplication level of the clean data were calculated. For FNG, the sequenced left files (read1 files) from two individuals were pooled into one big left.fq file, and right files (read2 files) into one big right.fq file. Transcriptome assembly was accomplished based on the left.fq and right.fq using Trinity program (trinityrnaseq_r20140413) with minimum k-mer coverage set to 2 and all other parameters set by default (Grabherr et al., 2011).

Functional annotations of all assembled unigenes were conducted by searching against the following databases: NCBI non-redundant protein (Nr), NCBI non-redundant nucleotide (Nt), Protein family (Pfam), Clusters of Orthologous Groups of proteins (KOG), Swiss-Prot protein (Swiss-Prot), KEGG Ortholog (KO), and Gene Ontology (GO) database.

### Orthologous Genes Identified and Phylogenetic Analysis

The phylogenetic tree of 18 species in Rosaceae was constructed with *Cannabis sativa* and *Morus notabilis* as outgroups. First, to define a set of conserved genes for cross-taxa comparison, we used OrthoMCL software (Li et al., 2003) to identify homologous gene clusters (orthogroups) among the twenty genomes. Genes with lengths less than 50 amino acids were excluded. OrthoMCL was run with an *e*-value cut-off of 1*e*-15 and an inflation parameter of 2.0. Then, orthogroups with only single copy genes (one-to-one orthologs) that were shared by all 20 genomes were retained for further analysis. Each orthogroups was aligned using MUSCLE v3.8.31 (Edgar, 2004) with default parameters. The poorly aligned regions were further strictly trimmed by using the trimAl v1.4 software (Capella-Gutiérrez et al., 2009) with the parameter “-gt 0.8 -st 0.001.” Alignments of all orthogroups were concatenated by our python script (**Supporting Information File S1**). Then maximum likelihood (ML) trees were generated using RAxML v7.0.4 (Stamatakis et al., 2005) with PROTCATJTT model, the ML criteria. The divergence time between all lineages of Rosaceae was estimated using the effectual newly developed MEGA7 software (Kumar et al., 2016). The timetree inferred using the Reltime method (Tamura et al., 2012) and the JTT matrix-based model (Jones et al., 1992). The timetree was computed using two clock calibration constraints. We calibrated with a 53.9–73.9 Mya divergence for Rosoideae vs. (Maloideae and Prunoideae) (Njuguna et al., 2013). The origin time of the common ancestor of Malus, Pyrus and Prunus has been dated to 28.7–56.1 Mya (Liu et al., 2014b).

### Positive Selection Analysis

Genome/transcriptome sequences of seven diploid *Fragaria* species and FAN were selected to conduct the positive selection analysis. The orthogroups of eight species were identified using the same methods which described in above phylogenetic analysis. In the positive selection analysis, only single copy genes shared by eight species were considered. We prepared the amino acid and codon-based nucleotide sequences in each orthogroups separately from eight species. The amino acid sequences of each orthogroups was aligned using MUSCLE v3.8.31 (Edgar, 2004) with default parameters. Then, to calculate the nonsynonymous (Ka) and synonymous (Ks) substitution rates between pairs of orthogroups, the corresponding multiple codon-based nucleotide alignment from the above aligned amino acid sequences were constructed by PAL2NAL (Suyama et al., 2006). For each alignment, a gene tree was constructed by RAxML (Stamatakis et al., 2005) using GTR+GAMMA model.

To estimate lineage-specific evolutionary rates for each branch of the eight species, the codeml program in the PAML 4 package (Yang, 2007) with the free-ratio model (model = 1) was run on each orthogroups. We conducted the boxplot analysis using the *K*a/*K*s ratio derived from free-ratio model results and filtered *K*a/*K*s < 0.0001 or *K*a/*K*s > 3. Significances of the deviations from the median *K*a/*K*s ratio between eight species branches were detected using Wilcoxon rank sum test. We also established frequency distribution plots of all *K*a/*K*s ratios of eight species.

To increase the power of our tests for positive selection, we applied the improved branch-site model (Zhang et al., 2005) implemented in codeml program (Yang, 2007) to estimate the *K*a/*K*s substitution rates (ω value). We also deleted all gaps (clean data = 1) from the alignments to lower the effect of ambiguous bases on the inference of positive selection. A foreground branch was specified as the clade of FAN. A significant likelihood ratio test (LRT) was conducted to determine whether positive selection is operating in the foreground branch. In this study, the extremely significant PSGs were inferred if the *P*-value was less than 0.01.

For PSGs in FAN, functional annotation was inferred by Gene Ontology (GO) and Kyoto Encyclopedia of Genes and Genomes (KEGG). GO enrichment analyses of PSGs were conducted using web-based agriGO^1^ (Du et al., 2010) with singular enrichment analysis (SEA) method and TAIR10 database. The KOBAS software (Xie et al., 2011) was also used to test the statistical enrichment of PSGs in KEGG pathways (Kanehisa and Goto, 2000).

## Results

### Transcriptome Assembly and Annotation

After quality filtering, we generated 59.29 (FVI) – 107.94 (FNG; Abbreviation of species names see **Table 1**) million clean reads for a total of 8.89 (FVI) – 13.48 (FNG) Gb of RNA-seq data from each of seven species (**Table 2**). The clean data were deposited in the NCBI Sequence Reads Archive (SRA) database (no. SRR4030219). *De novo* assembly of the high-quality reads generated 48,557 (FVI) – 82,537 (FNG) unigenes (the longest transcript in one gene; **Tables 1** and **2**). Median unigene lengths were 399 (FCO) – 459 (FMO) bp, with N50 lengths of 1,405 (FNG) – 1,678 (FVI) bp. There were 24,978 (FVI) – 35,166 (FNG) unigenes with homologs in Nr databases on the basis of similarity. A total of 30,825 (FVI) – 45,137 (FNG) unigenes were successfully annotated in at least one database (a total of seven databases, see Materials and Methods) with a significant match (*e*-value < 10^-5^; Supplementary Table S1). Most top species classification hit in the Nr database is *F. vesca* (>21,000 hits; Supplementary Table S2).

**Table 2:** Statistics of clean data and unigenes in this study.

Species	Statistics of clean data						Frequency distribution of unigenes’ lengths				
		
	Clean reads	Clean bases	Error (%)	Q20 (%)	Q30 (%)	GC (%)	Total number	Minimum Length	Median Length	Maximum Length	N50
FNG	107941470	13.48G	0.04	94.27	89.13	45.49	82537	201	412	16895	1405
FPE	68356166	10.25G	0.02	96.82	91.66	46.04	53748	201	443	16152	1655
FMA	80770294	12.12G	0.02	96.93	91.87	45.98	55911	201	435	16993	1672
FVI	59286810	8.89G	0.02	96.67	91.31	46.27	48557	201	456	15622	1678
FCO	83949596	12.59G	0.02	96.71	91.35	45.95	62587	201	399	16490	1563
FMO	71829414	10.77G	0.02	96.46	90.85	46.03	52413	201	459	15581	1655
FTI	86674308	13.0G	0.02	96.83	91.61	46.15	54022	201	449	15585	1638


### Phylogenetic Analysis and Divergence Date Estimation in Rosales

**FIGURE 1:**
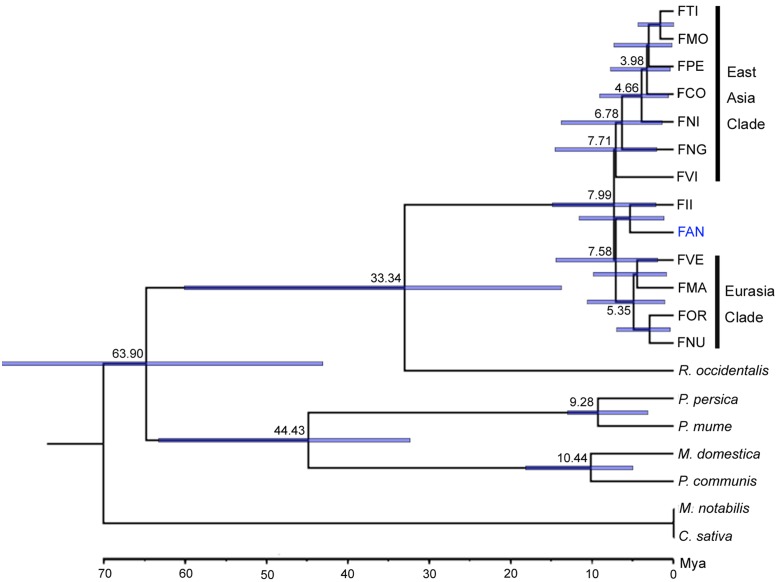
**Phylogenetics and divergence time among Rosales**.

Orthologs are genes that have evolved from a common ancestral gene via speciation (Fitch, 1970). Based on 20 genome and transcriptome sequences of Rosales, a total of 63,122 orthogroups were detected, including 515,610 genes. Among these, 4,690 orthogroups are shared by all species, and 276 orthogroups contain putative 1:1:1 single copy genes. Subsequently, we constructed a phylogenetic tree based on 87,916 amino acid sequences of the trimmed and concatenated 276 single copy gene alignments from 20 species (**Figure 1**). Using Reltime method and JTT matrix-based model, the estimated log likelihood value is -487560.5083. All positions with less than 90% site coverage were eliminated. There were a total of 63796 positions in the final dataset. The phylogenic tree shows that *Fragaria* closed grouped with *Rubus* and the *Fragaria* clade is divided into three subclades: an East Asian clade (FTI, FMO, FPE, FCO, FNG, and FVI), a Euro-Asian clade (FVE, FMA, FOR and FNU) and a cultivated strawberry clade (FAN and FII). Divergence time estimation suggests that *Fragaria* originated ∼7.99 Mya and split from Rubus ∼33.34 Mya, which combined diverged from the clade containing *Malus*, *Pyrus*, and *Prunus* ∼63.90 Mya. The species distributed in Southwest China (FTI, FMO, FPE, and FCO) split from the species in Japan (FNI) ∼4.66 Mya.

### Substitution Rates Estimated in Eight Strawberries

**FIGURE 2:**
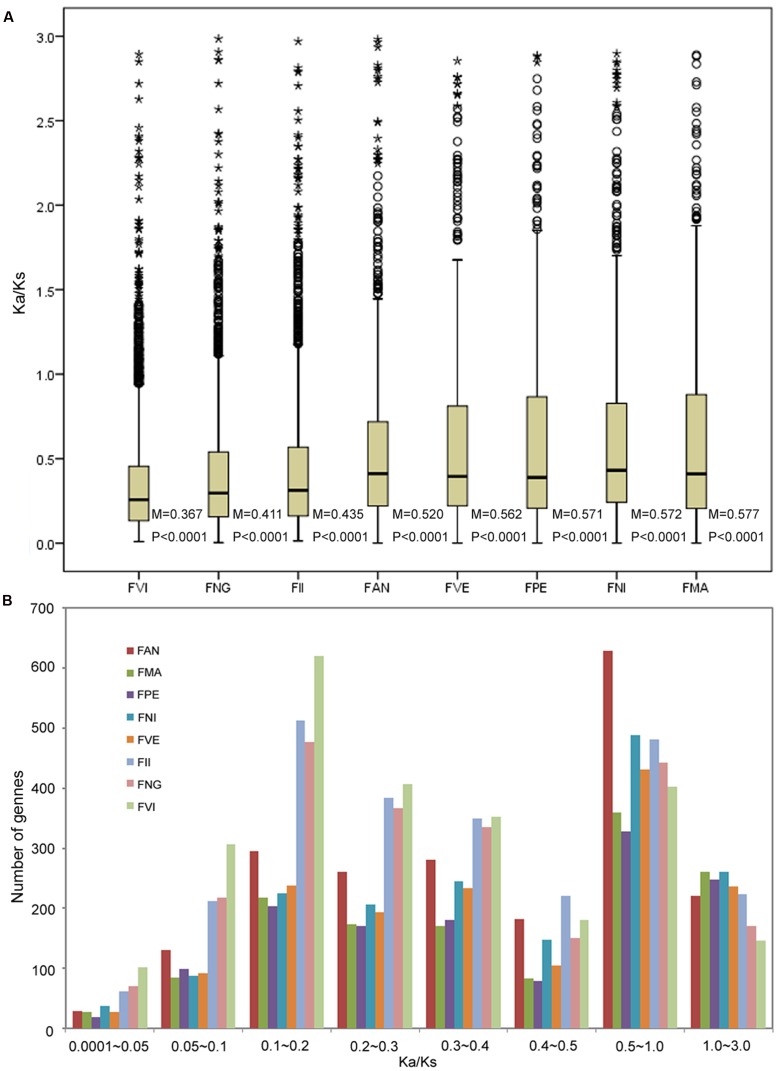
**Number of orthologs with given *dN*/*dS* ratios for the eight species.** **(A)** Boxplots of dN/dS ratios for each species. The median (M) and significances (P) of the deviations using Wilcoxon rank sum test are also showed. **(B)** Frequency distribution of dN/dS ratios of orthologs for the eight species.

Based on genome and transcriptome sequences of seven diploid *Fragaria* species and FAN, a total of 48,643 orthogroups were detected, including 240,023 genes. Among these, 8,593 orthogroups are shared by all eight species, and 4,487 orthogroups contain putative 1:1:1 single copy genes. Using these genes, we estimated the substitution rates for each orthogroup using the free-ratio model in PAML, which allows an independent *K*a/*K*s ratio for each branch (Yang, 2007). The boxplot result showed that the FVI has the smallest Ka/Ks median value (0.367), and the FMA has the biggest median (0.577). The median of FAN is 0.520, which adjacent with FII (0.435) and FVE (0.562). All the significance level of Wilcoxon rank sum between two neighboring species are less than 0.0001 (**Figure 2A**). For each species, most *K*a/*K*s ratios were in the ranges 0.1–0.2 and 0.5–1.0 (**Figure 2B**). The frequency distribution of *K*a/*K*s ratios shows that FAN has more genes (629, Supplementary Table S5) with elevated *K*a/*K*s ratios (1.0 > *K*a/*K*s > 0.5) than other species (328–489 genes; **Figure 2B**).

### Positive Selection Analysis in Cultivated Strawberry (*F. × ananassa*)

**FIGURE 3:**
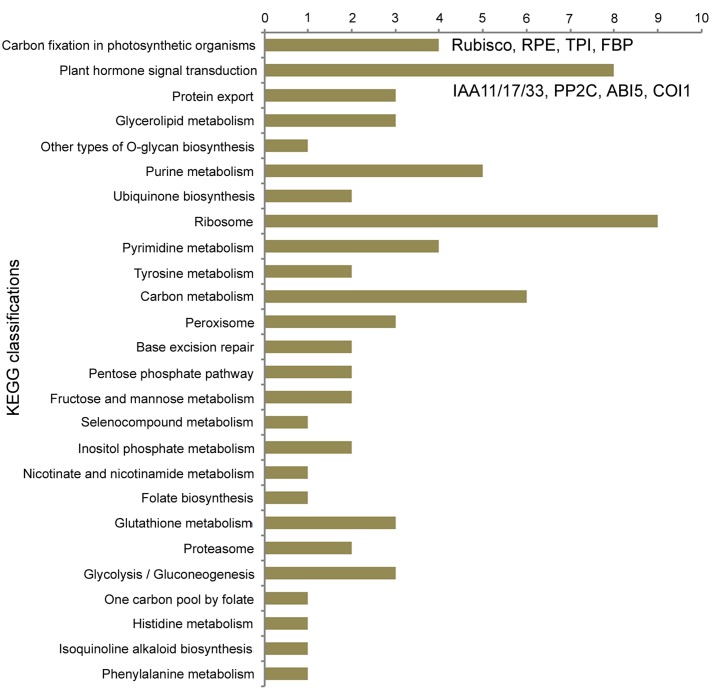
**Distribution of KEGG classifications of PSGs in FAN**.

For 4,487 single copy orthogroups in eight diploid species, the branch-site model of the PAML 4 package (Yang, 2007) was used to detect genes in FAN with signals of positive selection. As a result, 1,924 genes possibly under positive selection were identified in the FAN genome (*K*a/*K*s > 1); of these genes, 564 show significant evidence of positive selection (*P*-value < 0.05), and 510 PSGs show extremely significant evidence of having undergone positive selection (*P*-value < 0.01; Supplementary Table S3). We also conducted KEGG functional classification (**Figure 3**, Supplementary Table S4) for these PSGs in FAN. The distribution of KEGG classifications of PSGs shows that of all the annotated categories, metabolic processes have the most hits (24 PSGs; **Figure 3**). Carbon fixation in photosynthetic organisms was enriched, with four PSGs encoding ribulose bisphosphate carboxylase (Rubisco), ribulose-phosphate 3-epimerase (RPE), triosephosphate isomerase (TPI), and fructose-1,6-bisphosphatase (FBP). The biosynthesis of secondary metabolites was also enriched, with 13 genes, such as genes encoding 4-coumarate–CoA ligase-like 6, which participates in ubiquinone and flavanone biosynthesis. Moreover, the plant hormone signal transduction process was also enriched, with eight PSGs, including genes encoding auxin-responsive proteins IAA11, IAA17, and IAA31, protein phosphatase 2C (PP2C), abscisic acid-insensitive 5-like (ABI5-like) proteins, transcription factor TGA4-like isoform X1, and coronatine-insensitive protein 1 (COI1).

## Discussion

Our phylogenetic results based on nuclear genomes mostly agree with previous relationships determined using two nuclear genes or whole chloroplast genomes (Rousseau-Gueutin et al., 2009; Njuguna et al., 2013). The topology of major clades in the *Fragaria* genus is divided into three subclades with high support (100%), a Eurasian clade, an FII-FAN clade and an East Asian clade, which roughly correspond to clades A, B, and C, respectively, in the phylogenetic tree based on chloroplast genomes (Njuguna et al., 2013). East Asian species appear in all clades, indicating that this area is the center of origin of *Fragaria*, which is consistent with previous findings (Njuguna et al., 2013). However, there are some inconsistencies between our results and previous studies. The placement of FVI and FNG is grouped with East Asian clade with moderate support (70%) in our analysis, which has been unresolved in previous studies (Njuguna et al., 2013). In addition, FNU was incomprehensibly clustered with FOR in the Eurasian clade, which conflicts with the accepted idea that FNU is close to the East Asian clade (Rousseau-Gueutin et al., 2009). To be prudent, our natural selection analysis did not include this species’ genome sequence. Our analysis supported FAN clustering with FII rather than with Eurasian species. One reason might be that our analysis did not include the octoploid cross parents of cultivated FAN, i.e., *F. virginiana* and *F. chiloensis*, which most likely derived from diploid FVE or FMA by tetraploidization (Rousseau-Gueutin et al., 2009). Another reason is that FII is an important diploid donor of allopolyploid FAN according to resent studies, which indicated that four subgenomes of allo-octoploid cultivated strawberry derived from FVE, FII, and two with an unknown ancestor close to FII (Tennessen et al., 2014; Sargent et al., 2016). Since the virtual diploid genome of FAN was created by the overlap graph method, which is essentially a majority-rule consensus process, it should closely resemble the FII clade which provides the majority of the subgenomes. Therefore, it is understandable that our results show a close phylogenetic association between FAN and FII.

The appropriate calibration is important to the results of phylogenetic dating. In order to reduce the error, we have used two calibration constraints from two credible references (Njuguna et al., 2013; Liu et al., 2014b). From our dating results, we infer that the origin of *Fragaria* dates (∼7.99 Mya) from the Miocene, moderate earlier than the estimate (1.52–4.44 Mya) based on chloroplast genomes (Njuguna et al., 2013). The major reason might be that the genome sequences of the closest genus, *Potentilla*, were unavailable and not included in our analysis. Notably, the clade including four species (FTI, FMO, FPE, and FCO) found in the mountains of southwest China is inferred to have originated ∼3.98 Mya, which is in accordance with the time (3.6 Mya) of the strongest Qinghai–Tibet Plateau (QTP) rapid uplift in the Pliocene (Li and Fang, 1999). It suggests that the uplift of the QTP is a driving force for rapid speciation in this clade of Fragaria. That is, ecological adaptation has occurred within *Fragaria* following climatic and environmental changes after the QTP uplift, which promoted the speciation of these species. As has been reported for many other taxonomic groups, the QTP uplift is usually responsible for the initial isolation and subsequent climatic shifts and has driven the species divergence (Liu et al., 2012, 2014a; Zhou et al., 2012; Wen et al., 2014; Favre et al., 2015).

To investigate selective pressures in eight diploid species, we estimated substitution rates using a free model for each orthogroup. For all species, *K*a/*K*s ratios of most orthogroups were less than 0.5, indicating that purifying selection is the main driving force in *Fragaria* evolution, acting to remove deleterious mutations. The frequency distribution of *K*a/*K*s clearly showed that the cultivated species FAN has more genes with elevated interval values (Ka/Ks = 0.5–1.0) than the other seven species. The accelerated evolution of genes is often driven by positive selection, relaxed selection or low effective population size (Ne). During cultivation or domestication, both selection pressures for fitness traits important in the wild species and effective population size are reduced (Maceachern et al., 2009). Therefore, reduced selective constraint and reduced Ne could be responsible for the observed elevated evolutionary ratios in cultivated FAN with more elevated interval values (Ka/Ks = 0.5–1.0). While the similar number of positive selected genes (Ka/Ks > 1.0) between domesticated and wild *Fragaria* may be caused by artificial selection and natural selection respectively. This finding is compatible with branch-site model test for positive selection analysis, which indicated that many genes related to specific agronomic traits in FAN show significant positive selection. Moreover, the median of substitution rates (ka/ks ratio) of FAN is bigger than that of FII while smaller than FVE in free model (**Figure 2A**). It is noted that the substitution rates median of FAN is closer to FVE than to FII. As described above, the FAN is a virtual genome constructed from an allopolyploid via the overlap layout consensus method; we cannot rule out loci that evolved fast in FAN may actually be loci obtain from FVE rather than FII. Previous study also suggested that extensive unidirectional introgression has converted FII-like subgenomes to be more FVE-like among subgenomes (Tennessen et al., 2014). The reason might be homoploid hybridization in the FII-like diploid ancestors or strong selection spreading FVE-like sequence within the octoploid genome.

For a cultivated strawberry, many domestication-related genes in FAN involving traits desirable to humans should evolve rapidly under artificial selection, alongside natural selection caused by environmental factors. During the domestication process, fruit quality and flavor are the most important agronomic traits for strawberries. Carbohydrates, produced by photosynthesis in plants, are the basic substances of fruit growth and development and are closely related to fruit quality. In our KEGG enrichment analysis for PSGs in FAN, the “carbon fixation in photosynthetic organisms” pathway was the most significant hit. In this pathway, all four detected PSGs encode photosynthesis-related proteins (Rubisco, RPE, TPI and FBP). Rubisco is an essential and rate-limiting enzyme involved in the first step of carbon fixation and subsequently fixed into sugars (Chan et al., 1990). RPE catalyzes the reversible epimerization of D-ribulose 5-phosphate to D-xylulose 5-phosphate. TPI is involved in the gluconeogenesis and glycolysis pathways. Chloroplastic FBPase is a rate-limiting enzyme in the Calvin cycle, which plays a critical role in carbohydrate biosynthesis. A previous study reported that increasing the level of TPI and FBP aldolase in *Anabaena* could enhance photosynthetic activity and increase the rate of cell growth (Ma et al., 2007). We can speculate that as a trait desirable to humans, fruit quality, which can be described as the amount and composition of carbohydrates accumulated in the fruit, could undergo significant artificial selection during the domestication process. These key enzymes involved in carbon fixation and carbohydrate biosynthesis pathways show significantly positive selection further confirms this speculation.

Fruit quality and flavor are also closely associated with plant hormone biosynthesis and signal transduction (Obroucheva, 2014). Notably, the second most significantly enriched KEGG process in our results is plant hormone signal transduction, which includes eight PSGs in FAN. Among these PSGs, three homologs have similar functions in the auxin signaling process. These genes encode auxin-responsive proteins IAA11, IAA17, and IAA31, which function as repressors of early auxin response genes at low auxin concentrations (Liscum and Reed, 2002). A previous study based on an early stage fruit transcriptome in FVE revealed that *IAA31* was significantly up-regulated (6.5-fold) in seeds by comparing post-fertilization and pre-fertilization stages, which suggested that auxin exerts a direct role in stimulating receptacle expansion during fruit development (Kang et al., 2013). We speculate that these PSGs involved in auxin signaling might play specific functions in early fruit development in the cultivated strawberry.

The strawberry is a non-climacteric fruit, whose ripening is directly stimulated by abscisic acid (ABA) rather than ethylene (Jia et al., 2011; Obroucheva, 2014). ABA signaling via PYR/PYL receptors begins with PP2Cs, which permit activation of SNF1-related protein kinase 2, and then regulate downstream targets such as ABA-response transcription factors. The signaling pathway of ABA involves three PSGs, including two abscisic acid-insensitive 5-like (ABI5-like) proteins, and PP2C 37 (PP2CA). Among these, PP2CA acts as a key negative regulator of ABA responses to plant growth and development, inhibition of vegetative growth, and response to glucose (Yoshida et al., 2006; Park and Cutler, 2009). For example, PP2C is highly expressed during cucumber fruit development and may be involved in regulating fruit ripening (Wang et al., 2012). Two PSGs encode ABI5-like proteins, which participate in positively regulating ABA-mediated inhibition of seed germination and growth to promote fruit maturation (Brocard et al., 2002; Chen et al., 2008; Reeves et al., 2011). Previous study has also shown that exogenous sugar can significantly promote strawberry ripening while stimulating ABA accumulation (Jia et al., 2011). Therefore, all of the above PSGs are apparently related to fruit development and ripening, which attract major attention during domestication.

## Author Contributions

TZ, JL, YZ, JH, and HS conceived and designed the project. JL, LX, QQ, TZ, YZ, HS, and QW prepared samples. QQ, TZ, QW, and LX contributed to sequencing experiment. TZ and QQ performed data analyses. TZ, QQ, and JH wrote the paper. All authors read and approved the final manuscript.

## Conflict of Interest Statement

The authors declare that the research was conducted in the absence of any commercial or financial relationships that could be construed as a potential conflict of interest.

## Footnotes

1 bioinfo.cau.edu.cn/agriGO

## Supplementary Material

**FILE S1 | Python script for concatenate sequences of all orthogroups.**
