# Supplementary material for: Comparative Transcriptomics of Strawberries (Fragaria spp.) Provides Insights into Evolutionary Patterns
Source: Front Plant Sci. 2016 Dec 15;7:1839. doi: 10.3389/fpls.2016.01839 (PMC5156730; doi:10.3389/fpls.2016.01839)
Supplement: Supplementary file 1 [file Data_Sheet_1.PDF]

```
#!/usr/bin/env python
import sys
from Bio import SeqIO
if len(sys.argv) < 2:
        print "py in.1.fa in.2.fa ...."
        exit(1)


dic = {}
for i in sys.argv[1:]:
        for j in SeqIO.parse(i, 'fasta'):
        seqid    = j.id
        seqkey = seqid.split('|')[0]
        seqfa    = str(j.seq)
        if not dic.has_key(seqkey):

        else:


for i in dic:
        w = open('%s.fa' % i, 'w')
        w.writelines('%s\n%s\n' % ('_'.join(dic[i][0]), ''.join(dic[i][1])))
        w.close()
```
